# Successful Treatment of Concurrent Cholangiohydatidosis with Obstructive Jaundice and Hepatothoracic Transit in a Pediatric Patient

**DOI:** 10.1055/a-2590-5917

**Published:** 2025-05-10

**Authors:** Narcis Flavius Tepeneu, Călin Marius Popoiu, Emil Radu Iacob, Simona Cerbu, Oana Belei, Rodica Heredea

**Affiliations:** 1 Department of Pediatric Surgery and Orthopaedics, University of Medicine and Pharmacy “V. Babes”, Timisoara, Timisoara, Romania; 2 Department of Children's and Adolescent Surgery, Hospital Klagenfurt Am Wörthersee, Klagenfurt Am Wörthersee, Austria; 3 Pediatric Surgery and Orthopaedics Clinic, “L. Turcanu” Emergency Children's Hospital, Timisoara, Romania

**Keywords:** cholangiohydatidosis, hepatothoracic transit, echinococcus serology, ERCP

## Abstract

Concurrent rupture of hepatic hydatid cysts into the biliary tree and into the pleural cavity is a very rare complication in echinococcosis and can pose diagnostic and treatment challenges. We present the case of a 15-year-old female patient with recurrent abdominal pain, chest pain, fever, vomiting, jaundice, and cholangitis. Ultrasound, X-rays, computed tomography of the abdomen and thorax and cholangio-magnetic resonance imaging revealed a hepatic hydatid cyst with rupture into the main biliary duct causing obstruction, gallbladder microlithiasis, rupture of the right hemidiaphragm, and pleural hydatidosis. Echinococcus serology tests were positive. Endoscopic retrograde cholangiopancreatography (ERCP) could not resolve the obstructive jaundice. A laparotomy with choledochotomy, removal of hydatid structures, choledochal drainage with Kehr tube, cholecystectomy, Lagrot partial pericystectomy, partial pleural resection, suturing of the diaphragm, and triple drainage (right pleural cavity, cystic cavity, and Douglas pouch) was performed. Perioperative albendazole and antibiotic therapy was administered. The patient had an uneventful postoperative course. Follow-up at 1, 6, 12, and 24 months showed a favorable evolution without relapse of the hydatidosis. The very rare complications of cholangiohydatidosis and concomitant hepatothoracic transit lead to a severe condition, which needs adequate surgical treatment. Clinical presentation and laboratory findings are not specific and may simulate an obstructive jaundice and acute cholangitis of other etiology. ERCP with endoscopic papillotomy offers the advantage of a minimally invasive surgery, but it does not allow a definitive treatment of the whole problem and may be useful as a bridge procedure to drain the bile duct while awaiting definitive surgery.

## Introduction

Intrabiliary rupture of hepatic echinococcosis is an evolutionary complication of this pathological entity. Once this rupture occurs and there is communication between a cyst and the biliary tree, the conditions are created that allow the further migration of the parasitic structures (pieces of germinal layer or daughter vesicles) into the biliary tract. 1 2 This situation (cholangiohydatidosis) may produce a secondary cholangitis and even infection of the cyst with liver abscess.

Another evolutionary complication is hepatothoracic transit. It is an uncommon condition, which simultaneously involves the liver, diaphragm, and lung secondary to the migration of a hepatic hydatid cyst. Its estimated prevalence is between 2 and 11% in adults. 3 4

It is a difficult clinical condition to treat due to, among other things, the cyst in transit sometimes being infected, producing a secondary hepatic abscess, 5 6 or it is in direct communication with the bronchial tree, which manifests as a cough, dyspnea, thoracic pain, and possibly biliptysis.

There may also be coexistent cholangiohydatidosis. 7 8 All these situations can exacerbate the risk of postoperative morbidity (POM). 6 8

## Case Report

We present the case of a 15-year-old female patient with recurrent abdominal pain, chest pain, fever, vomiting, jaundice, and cholangitis. The symptoms started about 2 weeks prior to presentation in the hospital. Obstructive jaundice markers and inflammatory markers were elevated ( Table 1 ).

**Table 1:** Preoperative laboratory data

Parameter	Value	Normal value
Bilirubin (direct)	19.56 µmol/L	<5 µmol/L
Bilirubin (total)	25.45 µmol/L	<17 µmol/L
WBC	31,790/µL	4,800–10,800/µL
Hb	8.7 g/dL	11.8–15.7 g/dL
Ht	28.3%	34–45%
PLT	700,000/µL	150,000–450,000/µL
ALT	217 U/L	<23 U/L
AST	214 U/L	<27 U/L
GGT	236 U/L	5–40 U/L
ESR	167.76 mm/h	2–13 mm/h
CRP	121.74 mg/dL	0–5 mg/dL
ALP	557 U/L	5–40 U/L
Fibrinogen	487 mg/dL	180–390 mg/dL
Ac anti-HCV	Negative	Negative
Atg Hbs	Negative	Negative
HIV 1 and 2	Negative	Negative
Lipase	30 U/L	13–60 U/L
Creatinine	39 µmol/L	44–80 µmol/L
Urea	3.06 mmol/L	1.4–8.03 mmol/L
Urine test	Normal, except 15 leukocytes/µL	0–10 L/µL
Urine culture	Negative	Negative

Abbreviations: ALP, alkaline phosphatase; ALT, alanine aminotransferase; AST, aspartate aminotransferase; Atg Hbs, hepatitis B surface antigen; CRP, C-reactive protein; ESR, erythrocyte sedimentation rate; GGT, gamma-glutamyl transferase; Hb, hemoglobin; HCV, hepatitis C virus; HIV, human immunodeficiency virus; Ht, hematocrit; PLT, platelet; WBC, white blood cell count.

An ultrasound of the abdomen revealed a hepatic cyst in the right hepatic lobe (segment VII) with dimensions of 6.7/6.8/6.8 cm with dilated common bile duct (1.7 cm in diameter), gallbladder hydrops, and gallstone of 6.5 mm. A thoracic X-ray showed no anomalies. With the suspicion of a disseminated echinococcosis a computed tomography of the abdomen and thorax was performed. The investigation showed a disseminated hepatic hydatid cyst with cholangiohydatidosis with obstructive jaundice and hepatothoracic transit into the right pleural space through the diaphragm and a gallstone. Anti-Echinococcus IgG antibodies were positive.

**Fig. 1:**
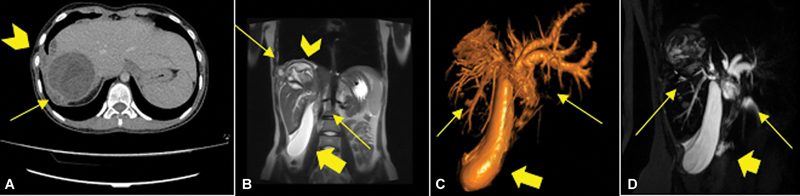
( **A** ) CT image showing the hydatic hepatic cyst (arrow) with hepatothoracic transit (arrowhead). ( **B, D** ) Preoperative cholangio-MRI showing the ruptured hepatic hydatid cyst in segment VII with subsequent cholangiohydatidosis (arrows), dissemination of the hydatid cyst, gallbladder stones and hydrops (thick arrow) and hepatothoracic transit (arrowhead). ( **C** ) Preoperative cholangio-MRI showing a 3D image reconstruction of the biliary tree with the ruptured hepatic hydatid cyst in segment VII with subsequent cholangiohydatidosis (arrows) and hydrops (thick arrow). 3D, three-dimensional; CT, computed tomography; MRI, magnetic resonance imaging.

An endoscopic retrograde cholangiopancreatography (ERCP) with endoscopic papillotomy tried to clear out the hydatid cyst debris from the common bile duct but was unsuccessful in relieving the symptoms. A cholangio-magnetic resonance imaging (MRI) revealed that the germinative hydatid cyst membrane had prolapsed into the common bile duct with subsequent cholangiohydatidosis, obstructive jaundice, dissemination of the hydatid cyst, gallbladder stones, hydrops, and hepatothoracic transit ( Fig. 1A–D ).

A laparotomy with choledochotomy, removal of hydatid structures, choledochal drainage with Kehr tube, cholecystectomy, Mabbit–Lagrot partial pericystectomy, partial pleural resection, suturing of the diaphragm, and triple drainage (right pleural cavity, cystic cavity, and Douglas pouch) was performed.

The procedure was performed through a transabdominal approach (Kocher right subcostal incision, which was slightly extended to the left). The partial resection of the right pleura was done transdiaphragmatically, including the affected portion of the diaphragm. Because of the anatomy and the location of the hydatid cyst and the extension through the diaphragm into the right pleura we decided to resect the cyst with the affected portion of diaphragm and pleura.

Dissemination of protoscolices-rich fluid during surgery for hydatid cyst disease is a major cause of recurrence. Instillation of a scolicidal agent into a hepatic hydatid cyst before opening is the most commonly employed measure to prevent this serious complication. We used 20% hypertonic saline to inactivate the parasite.

The diaphragm was repaired with nonabsorbable sutures and the right pleural space was drained through the sixth intercostal space.

Perioperative albendazole and antibiotic therapy was administered.

The Kehr tube was kept in place for 25 days after the operation. The right pleural drain was kept in place for 6 days and the drain in the remaining cystic cavity for 18 days.

The total hospitalization period was 43 days.

**Fig. 2:**
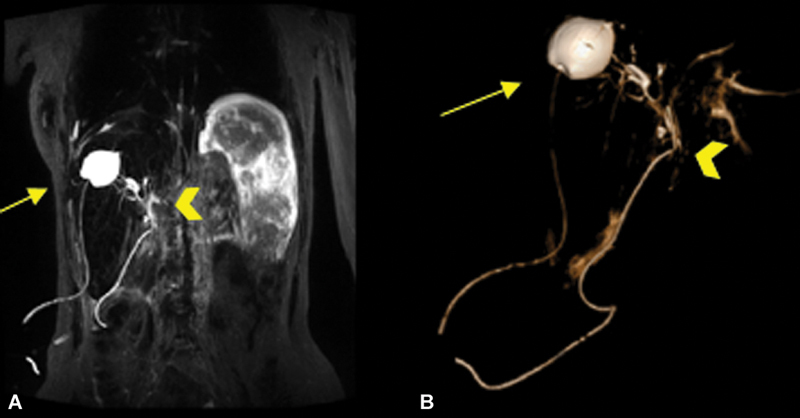
( **A** ) Postoperative cholangio-MRI showing the drainage tube in the restant cystic cavity after Mabbit–Lagrot partial pericystectomy (arrow) and the Kehr biliary tube in the common bile duct (arrowhead). ( **B** ) Postoperative cholangio-MRI showing a 3D reconstruction with the drainage tube in the restant cystic cavity after Mabbit–Lagrot partial pericystectomy (arrow) and the Kehr biliary tube in the common bile duct (arrowhead). 3D, three-dimensional; MRI, magnetic resonance imaging.

The patient had an uneventful postoperative course ( Fig. 2A, B ).

Follow-up at 1, 6, 12, and 24 months showed a favorable evolution without relapse of the hydatidosis.

## Discussion

Concurrent rupture of hepatic hydatid cysts into the biliary tree and into the pleural cavity is a very rare complication in echinococcosis and can pose diagnostic and treatment challenges.

In addition to being a rare entity, the biggest problems are the complications that cholangiohydatidosis can cause: biliary obstructive syndrome, 9 10 acute suppurated cholangitis, 9 10 11 and even acute secondary pancreatitis. 10 12

Clinical presentation and laboratory findings are not specific and may simulate an obstructive jaundice and acute cholangitis of other etiology. 13 14

Treatment of cholangiohydatidosis and secondary cholangitis is to drain and clean the bile duct, which can be performed by open (conventional) surgery or closed surgery (ERCP with endoscopic papillotomy). However, while it is true that endoscopic papillotomy offers all the advantages of a minimally invasive surgery, it does not allow a rational and definitive treatment of the whole problem. Therefore, in general, surgery remains the option of choice through biliary drainage (choledochostomy or choledocoduodenoanastomosis) and simultaneous resection of the hydatid cyst. 13 14 15

ERCP with papillotomy without stenting was the first choice for clearing the common bile duct from the hydatid cyst debris in our case. The problem with ERCP was that it tried to clear out the hydatid cyst debris from the common bile duct but was unsuccessful in keeping the common bile duct patent, and in relieving the symptoms. The subsequent cholangio-MRI revealed that the germinative hydatid cyst membrane had prolapsed into the common bile duct. Stenting was not done, but we suspect that it would not have solved the problem either, because it would not have allowed the passage of the germinative hydatid cyst membrane because of its dimensions.

Also in the case we present there was hepatothoracic transit.

There is controversy surrounding the ideal surgical access to treat hepatothoracic transit—thoracic, abdominal, or thoracoabdominal. 16 17 The technique should depend on the location of the lesion, the condition and size of the cyst, and the experience of the surgical team.

In our case the procedure was performed through a transabdominal approach (Kocher right subcostal incision, which was slightly extended to the left), which allowed treatment of the whole problem.

Regarding using Mabbit–Lagrot procedure, we chose this procedure for treatment of the hepatic hydatid cyst because of the following reasons:

1. We have a vast and positive experience in our clinic with this technique (experience over 50 years of time).
2. This is a complex case with rupture of the hydatid cyst in the main bile duct. In this case surgical treatment must be as conservative as possible in order to reduce the mortality and morbidity rate of this condition.

Alternatives cited in literature in this case are: suture of the cystobiliary fistula, catheterization, Pomodoro technique, drainage of the main bile duct, biliodigestive anastomosis, total pericystectomy, hepatic resection(partial or anatomical), cholecystectomy with bile duct exploration, ERCP, endoscopic sphincterotomy, biliary stent placement, basket or balloon extraction of cyst fragments. 8 9 18 19

Dissemination of protoscolices-rich fluid during surgery for hydatid cyst disease is a major cause of recurrence. Instillation of a scolicidal agent into a hepatic hydatid cyst before opening is the most commonly employed measure to prevent this serious complication.

We used 20% hypertonic saline, which has in the literature a moderate risk for biliary damage, but we did not observe any problems with the use of this substance in hydatid cyst surgery in numerous cases of intra-abdominal hydatidosis (liver, spleen, pancreas, ovary, etc.) operated in our clinic in the long-term follow-up. There is a higher morbidity risk in higher concentrations like 30% NaCl, but we never use that.

High concentrated sodium solutions are contraindicated in biliary fistulas primarily because they can cause chemical irritation and damage to the biliary epithelium. While this could potentially lead to sclerosing cholangitis as a complication, it is not the main reason for the contraindication.

The main concerns with using high concentrated sodium solutions in biliary fistulas are:

1. Direct chemical injury to the bile duct epithelium.
2. Risk of local tissue necrosis.
3. Potential for creating inflammatory reactions.
4. Possibility of worsening the existing fistula.

Sclerosing cholangitis could develop as a secondary complication due to the inflammatory and scarring processes triggered by the chemical irritation, but it is more of a potential consequence rather than the primary reason for avoiding these solutions.

With respect to medical treatment with albendazole, there is strong evidence (systematic reviews and clinical trials) that indicate that anthelmintic treatment alone is not ideal for liver hydatid cysts. 18 19 20 On the other hand, the World Health Organization carried out multicenter trials in Europe to compare albendazole and mebendazole and verified that both drugs had similar efficacy, but mebendazole required higher doses and a different treatment with longer periods of adverse reactions. 21 22

## Conclusion

The very rare complications of cholangiohydatidosis and concomitant hepatothoracic transit lead to a severe condition, which needs adequate surgical treatment. Clinical presentation and laboratory findings are not specific and may simulate an obstructive jaundice and acute cholangitis of other etiology. ERCP with endoscopic papillotomy offers the advantage of a minimally invasive surgery, but it does not allow a definitive treatment of the whole problem and may be useful as a bridge procedure to drain the bile duct while awaiting definitive surgery.

Finally, it is important to note that the association of cholangiohydatidosis with hepatothoracic transit is a rare cause of obstructive jaundice and cholangitis associated with important rates of POM and mortality, which occurs in the context of complex patients, so it must be treated in centers with experience in such diseases.

## Notes

New Insights and the Importance for the Pediatric Surgeon Concurrent rupture of hepatic hydatid cysts into the biliary tree and into the pleural cavity is a very rare complication in echinococcosis and can pose diagnostic and treatment challenges.
